# Prescribing patterns in older people with advanced chronic kidney disease towards the end of life

**DOI:** 10.1093/ckj/sfae301

**Published:** 2024-10-04

**Authors:** Matthew Letts, Nicholas C Chesnaye, Maria Pippias, Fergus Caskey, Kitty J Jager, Friedo W Dekker, Merel van Diepen, Marie Evans, Claudia Torino, Antonio Vilasi, Maciej Szymczak, Christiane Drechsler, Christoph Wanner, Barnaby Hole, Samantha Hayward, the EQUAL study investigators, Andreas Schneider, Anke Torp, Beate Iwig, Boris Perras, Christian Marx, Christiane Drechsler, Christof Blaser, Christoph Wanner, Claudia Emde, Detlef Krieter, Dunja Fuchs, Ellen Irmler, Eva Platen, Hans Schmidt-Gürtler, Hendrik Schlee, Holger Naujoks, Ines Schlee, Sabine Cäsar, Joachim Beige, Jochen Röthele, Justyna Mazur, Kai Hahn, Katja Blouin, Katrin Neumeier, Kirsten Anding-Rost, Lothar Schramm, Monika Hopf, Nadja Wuttke, Nikolaus Frischmuth, Pawlos Ichtiaris, Petra Kirste, Petra Schulz, Sabine Aign, Sandra Biribauer, Sherin Manan, Silke Röser, Stefan Heidenreich, Stephanie Palm, Susanne Schwedler, Sylke Delrieux, Sylvia Renker, Sylvia Schättel, Theresa Stephan, Thomas Schmiedeke, Thomas Weinreich, Til Leimbach, Torsten Stövesand, Udo Bahner, Wolfgang Seeger, Adamasco Cupisti, Adelia Sagliocca, Alberto Ferraro, Alessandra Mele, Alessandro Naticchia, Alex Còsaro, Andrea Ranghino, Andrea Stucchi, Angelo Pignataro, Antonella De Blasio, Antonello Pani, Aris Tsalouichos, Bellasi Antonio, Biagio Raffaele Di Iorio, Butti Alessandra, Cataldo Abaterusso, Chiara Somma, Claudia D'alessandro, Claudia Torino, Claudia Zullo, Claudio Pozzi, Daniela Bergamo, Daniele Ciurlino, Daria Motta, Domenico Russo, Enrico Favaro, Federica Vigotti, Ferruccio Ansali, Ferruccio Conte, Francesca Cianciotta, Francesca Giacchino, Francesco Cappellaio, Francesco Pizzarelli, Gaetano Greco, Gaetana Porto, Giada Bigatti, Giancarlo Marinangeli, Gianfranca Cabiddu, Giordano Fumagalli, Giorgia Caloro, Giorgina Piccoli, Giovanbattista Capasso, Giovanni Gambaro, Giuliana Tognarelli, Giuseppe Bonforte, Giuseppe Conte, Giuseppe Toscano, Goffredo Del Rosso, Irene Capizzi, Ivano Baragetti, Lamberto Oldrizzi, Loreto Gesualdo, Luigi Biancone, Manuela Magnano, Marco Ricardi, Maria Di Bari, Maria Laudato, Maria Luisa Sirico, Martina Ferraresi, Michele Provenzano, Moreno Malaguti, Nicola Palmieri, Paola Murrone, Pietro Cirillo, Pietro Dattolo, Pina Acampora, Rita Nigro, Roberto Boero, Roberto Scarpioni, Rosa Sicoli, Rosella Malandra, Silvana Savoldi, Silvio Bertoli, Silvio Borrelli, Stefania Maxia, Stefano Maffei, Stefano Mangano, Teresa Cicchetti, Tiziana Rappa, Valentina Palazzo, Walter De Simone, Anita Schrander, Bastiaan van Dam, Carl Siegert, Carlo Gaillard, Charles Beerenhout, Cornelis Verburgh, Cynthia Janmaat, Ellen Hoogeveen, Ewout Hoorn, Friedo Dekker, Johannes Boots, Henk Boom, Jan-Willem Eijgenraam, Jeroen Kooman, Joris Rotmans, Kitty Jager, Liffert Vogt, Maarten Raasveld, Marc Vervloet, Marjolijn van Buren, Merel van Diepen, Nicholas Chesnaye, Paul Leurs, Pauline Voskamp, Peter Blankestijn, Sadie van Esch, Siska Boorsma, Stefan Berger, Constantijn Konings, Zeynep Aydin, Aleksandra Musiała, Anna Szymczak, Ewelina Olczyk, Hanna Augustyniak-Bartosik, Ilona Miśkowiec-Wiśniewska, Jacek Manitius, Joanna Pondel, Kamila Jędrzejak, Katarzyna Nowańska, Łukasz Nowak, Maciej Szymczak, Magdalena Durlik, Szyszkowska Dorota, Teresa Nieszporek, Zbigniew Heleniak, Andreas Jonsson, Anna-Lena Blom, Björn Rogland, Carin Wallquist, Denes Vargas, Emöke Dimény, Fredrik Sundelin, Fredrik Uhlin, Gunilla Welander, Isabel Bascaran Hernandez, Knut-Christian Gröntoft, Maria Stendahl, Maria Svensson, Marie Evans, Olof Heimburger, Pavlos Kashioulis, Stefan Melander, Tora Almquist, Ulrika Jensen, Alistair Woodman, Anna McKeever, Asad Ullah, Barbara McLaren, Camille Harron, Carla Barrett, Charlotte O'Toole, Christina Summersgill, Colin Geddes, Deborah Glowski, Deborah McGlynn, Dympna Sands, Fergus Caskey, Geena Roy, Gillian Hirst, Hayley King, Helen McNally, Houda Masri-Senghor, Hugh Murtagh, Hugh Rayner, Jane Turner, Joanne Wilcox, Jocelyn Berdeprado, Jonathan Wong, Joyce Banda, Kirsteen Jones, Lesley Haydock, Lily Wilkinson, Margaret Carmody, Maria Weetman, Martin Joinson, Mary Dutton, Michael Matthews, Neal Morgan, Nina Bleakley, Paul Cockwell, Paul Roderick, Phil Mason, Philip Kalra, Rincy Sajith, Sally Chapman, Santee Navjee, Sarah Crosbie, Sharon Brown, Sheila Tickle, Suresh Mathavakkannan, Ying Kuan

**Affiliations:** Population health sciences, Bristol Medical School, University of Bristol, Bristol, UK; Renal Unit, Southmead Hospital, North Bristol NHS Trust, Bristol, UK; ERA registry, Department of Medical Informatics, Amsterdam UMC, location Academical Medical Center, Amsterdam, The Netherlands; Amsterdam Public Health Research Institute, Quality of Care, Amsterdam, The Netherlands; Population health sciences, Bristol Medical School, University of Bristol, Bristol, UK; Renal Unit, Southmead Hospital, North Bristol NHS Trust, Bristol, UK; Population health sciences, Bristol Medical School, University of Bristol, Bristol, UK; Renal Unit, Southmead Hospital, North Bristol NHS Trust, Bristol, UK; ERA registry, Department of Medical Informatics, Amsterdam UMC, location Academical Medical Center, Amsterdam, The Netherlands; Amsterdam Public Health Research Institute, Quality of Care, Amsterdam, The Netherlands; Department of Clinical Epidemiology, Leiden University Medical Center, Leiden, The Netherlands; Department of Clinical Epidemiology, Leiden University Medical Center, Leiden, The Netherlands; Renal Unit, Department of Clinical Intervention and Technology (CLINTEC), Karolinska Institutet and Karolinska University hospital, Stockholm, Sweden; Institute of Clinical Physiology – National Research Council, Clinical Epidemiology and Pathophysiology of Renal Diseases and Hypertension, Reggio Calabria, Italy; Institute of Clinical Physiology – National Research Council, Clinical Epidemiology and Pathophysiology of Renal Diseases and Hypertension, Reggio Calabria, Italy; Department of Nephrology and Transplantation Medicine, Wroclaw Medical University, Wroclaw, Poland; Division of Nephrology, University Hospital of Würzburg, Würzburg, Germany; Division of Nephrology, University Hospital of Würzburg, Würzburg, Germany; Population health sciences, Bristol Medical School, University of Bristol, Bristol, UK; Renal Unit, Southmead Hospital, North Bristol NHS Trust, Bristol, UK; Population health sciences, Bristol Medical School, University of Bristol, Bristol, UK

**Keywords:** CKD, death, deprescriptions, elderly, polypharmacy

## Abstract

**Background:**

Advancing age and chronic kidney disease (CKD) are risk factors for polypharmacy. Polypharmacy is associated with negative healthcare outcomes. Deprescribing, the systematic rationalization of potentially inappropriate medications, is a proposed way of addressing polypharmacy. The aim of this study was to describe longitudinal prescribing patterns of oral medications in a cohort of older people with advanced CKD in their last years of life.

**Methods:**

The European QUALity (EQUAL) study is a European, prospective cohort study of people ≥65 years with an incident estimated glomerular filtration rate (eGFR) of ≤20 mL/min/1.73 m^2^. We analysed a decedent subcohort, using generalized additive models to explore trends in the number and types of prescribed oral medications over the years preceding death.

**Results:**

Data from 563 participants were analysed (comprising 2793 study visits) with a median follow-up time of 2.2 years (interquartile range 1.1–3.8) pre-death. Participants’ numbers of prescribed oral medications increased steadily over the years approaching death—7.3 (95% confidence interval 6.9–7.7) 5 years pre-death and 8.7 (95% confidence interval 8.4–9.0) at death. Over the years pre-death, the proportion of people prescribed (i) proton-pump inhibitors and opiates increased and (ii) statins, calcium-channel blockers and renin–angiotensin–aldosterone system inhibitors decreased, whilst (iii) beta-blockers, diuretics and gabapentinoids remained stable. At their final visits pre-death 14.6% and 5.1% were prescribed opiates and gabapentinoids, respectively.

**Conclusion:**

Elderly people with advanced CKD experienced persistent and increasing levels of polypharmacy as they approached the end of life. There was evidence of cessation of certain classes of medications, but at a population level this was outweighed by new prescriptions. This work highlights the potential for improved medication review in this setting to reduce the risks associated with polypharmacy. Future work should focus at the individual patient–clinician level to better understand the decision-making process underlying the observed prescribing patterns.

## Graphical Abstract

**Graphical Abstract:**
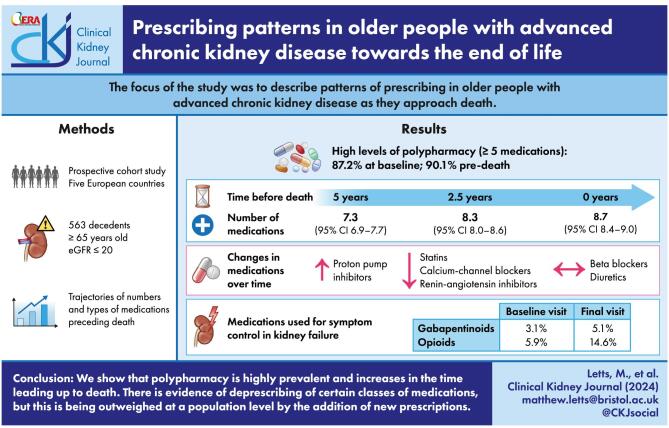

KEY LEARNING POINTS **What was known:**

- Advancing age and chronic kidney disease (CKD) are risk factors for polypharmacy.
- Polypharmacy is associated with adverse health outcomes.
- Deprescribing is a way of addressing polypharmacy.
- Patterns of prescribing in older people with advanced CKD as they approach the end of life are currently unknown.

**This study adds:**

- This is the first study to examine longitudinal prescribing trends in older people with advanced CKD.
- Polypharmacy is highly prevalent, and increases as people approach death.
- In this cohort there is evidence of deprescribing of certain medications, but this is outweighed at a population level by new prescriptions.

**Potential impact:**

- This study has described the trends in the number and types of medications prescribed to this cohort.
- It demonstrates to clinicians and researchers the current extent of polypharmacy, and guides which medications should be targeted when examining the reasons for prescribing and deprescribing at the individual patient–clinician level.

## INTRODUCTION

Longer life expectancy, increasing multi-morbidity, and the greater use of guideline-directed therapies, has led to people taking more medications [1, 2]. Polypharmacy (often defined as the regular intake of ≥5 medications) is particularly prevalent in the elderly [3], and those in the last years of life [4, 5]. It is associated with increased treatment burden, higher rates of drug–drug interactions, medication non-adherence, falls and hospitalizations, and a reduction in a person's function and quality of life [6–8]. It is well established that people with chronic kidney disease (CKD) often experience polypharmacy [9–11], and this is theorized to be due to their complex combination of comorbidities, CKD-related disorders (e.g. mineral bone disease and anaemia) and symptom burden [9].

The World Health Organization has identified polypharmacy as one of three ‘key action areas’ in its global effort to reduce medication-related harm [12]. Deprescribing (the systematic review and rationalization of a person's medications) has been studied as a potential way to reduce polypharmacy and improve health-related outcomes, particularly in older age [13, 14]. Specific medications have been identified as potentially inappropriate medications for older people with CKD [15, 16] and some of these have been targeted by previous deprescribing interventions [17–19]. Many in this group are approaching the ends of their lives and preventative medications may not be taken for long enough to convey their intended benefits; therefore, they can often be safely deprescribed whilst continuing with symptomatic treatments [20, 21]. There is no universal definition of a timeframe that constitutes the end of life, but this period can be analysed by retrospectively examining the years leading up to death in people who have died [22].

To our knowledge, no previous longitudinal analysis has been performed looking at prescribing patterns in the last years of life for an elderly population with advanced CKD. This work aimed to describe such patterns in a European population, and to examine for evidence of prescribing and deprescribing as people approach death.

## MATERIALS AND METHODS

### Study population

To examine prescribing trends in the last years of life, we analysed data from decedents in the European QUALity (EQUAL) study [23]. EQUAL is an ongoing international, multi-centre, observational, prospective cohort study which recruits people ≥65 years old with advanced CKD, from nephrology clinics in six countries: Germany, Italy, the Netherlands, Poland, Sweden and the UK. To be eligible for inclusion, a participant must have an incident estimated glomerular filtration rate (eGFR) ≤20 mL/min/1.73 m^2^ (as calculated by the Modification of Diet in Renal Disease equation) within the preceding 6 months. Participants are not eligible for inclusion if the drop in eGFR is due to an acute event, or if the person is in receipt of kidney replacement therapy (dialysis or transplant). The EQUAL study was approved by medical ethics committees in each of the participating countries, and participants provide written consent for inclusion.

For the current study, we selected all participants whose first study visit was between 1 March 2012 and 31 August 2021. A decedent subcohort was derived comprising the study participants who died during the study follow-up period. Participants from Sweden were excluded, as their medication data were obtained via linkage to the Swedish Renal Registry, and thus data on only a limited number of medications was collected.

### Data collection

Demographic, clinical and medication data were collected at the baseline study visit, and subsequently at 3- to 6-month intervals, by a research nurse using a standardized case record template, with information corroborated against medical records. Primary renal disease was defined according to the European Renal Association coding system for Primary Renal Disease [24]; the EQUAL investigators created standardized education categories to compare educational attainment across countries. The Charlson Comorbidity Index was used to calculate a weighted comorbidity score [25].

### Medication data

At each study visit, a complete list of the names of a participant's current prescribed medications was collected. Information on medication dosage, frequency, route and adherence were not recorded, nor were ‘over-the-counter’ preparations. For this analysis, study visits were excluded due to missing or duplicated medication data, or on the rare occasion when medications were recorded after the date of a participant's death.

All medications were coded according to their exact substance level Anatomical Therapeutic Chemical (ATC) code [26]. We excluded all anti-infectives due to their episodic nature, and all non-oral medications, as there was greater potential variability in how these were recorded; in addition these are less often targets for deprescribing. The ATC index states all administration routes for any given medication; non-oral medications were defined as those that have no available oral preparation. The total number of prescribed oral medications (POMs) at each study visit was calculated by taking a count of all of the recorded medications. Combination medications were counted as a single medication. For a list of all the names and frequences of prescribed medications, and whether they were included in our analyses, see Supplementary data, Table S1.

### Statistical analysis

The change in prescribing patterns over time was explored using Generalized Additive Models (GAMs) and the *mgcv* package in R [27]. GAMs can be seen as extensions of traditional generalized linear models, which allow for the modelling of non-linear relationships by replacing the linear (or other parametric) coefficients with flexible ‘smooth’ functions. In all analyses, time was anchored (t = 0) at death, and inverted back (in negative years) to the date of a participant's first study visit. We used ‘random effects smooths’, allowing the data to define the number of basis functions, and using the method of restricted maximum likelihood to define the smoothing parameter. This allows the data itself to determine the model's shape and ‘wiggliness’. We modelled the intercept and slope as random effects to allow for individual level variations in medication status at baseline and over time respectively.

The initial analysis explored the relationship between time (before death) and the total number of POMs. Pre-specified univariable stratifications were carried out by sex, baseline age, diabetes status (including and excluding medications for the treatment of diabetes), country of origin, baseline Charlson Comorbidity Index, and the presence or absence of a diagnosis of heart failure and coronary artery disease. To explore the effect of dialysis status on the number of POMs, data from participants who did not start dialysis were compared with those who did; for this latter group, only visits whilst on dialysis were included. These count data were modelled with the Poisson response distribution (and default log link function). As sensitivity analyses, the initial model was re-run with all medications included except anti-infectives, and using data only from participants who had ≥3 study visits, and ≥1 year of follow up data.

Subsequent analyses explored the relationship between time (before death) and the proportion of people prescribed specific classes of POMs. Specific classes were chosen to explore: (i) the extent of deprescribing of medications identified as potentially inappropriate in older people with CKD—statins and proton-pump inhibitors (PPIs) [15, 16]; (ii) patterns of prescribing of medications used for the control of symptoms of advanced CKD (as identified in the Global Kidney Health Atlas)—gabapentinoids and opiates [28]; (iii) the longitudinal trends of any other POM given to >25% of the cohort at baseline—allopurinol, aspirin, beta-blockers, calcium channel blockers (CCBs), diuretics, renin–angiotensin–aldosterone system inhibitors (RAASis), sodium bicarbonate and vitamin D [11]. These proportional data were modelled with the binomial response distribution (and default logit link function). Supplementary data, Table S2 details which medications (and ATC codes) were included in each of these class analyses.

To investigate prescribing patterns at the individual level, we compared the baseline visit and last visit pre-death of each decedent who had >1 study visit. The difference in the total number of POMs was calculated, then each of the previously defined specific POM classes were categorized as newly prescribed (not prescribed at first visit, but prescribed at last), deprescribed (prescribed at first visit, but not at last), never prescribed (not prescribed at first or last visits) or always prescribed (prescribed at first and last visits).

All analyses were carried out using R Statistical Software (v4.2.1) [29].

## RESULTS

### Population characteristics

**Figure 1:**
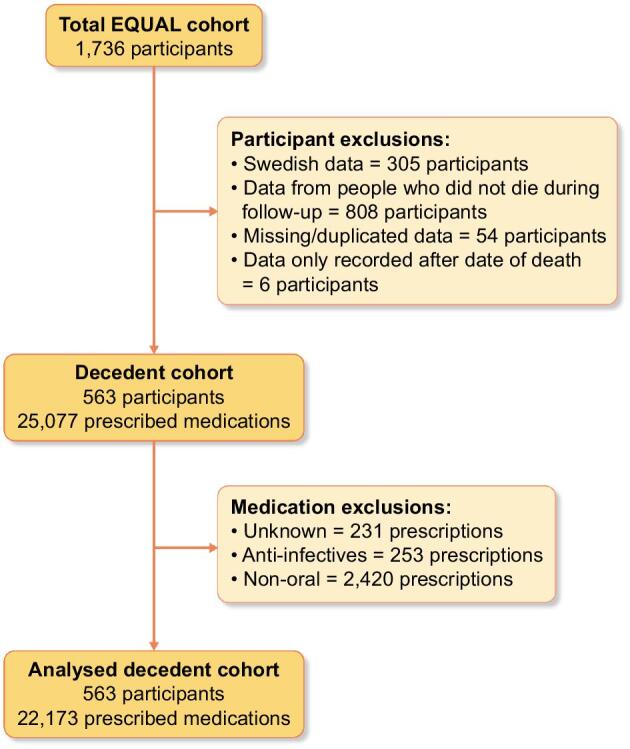
Cohort flow diagram showing numbers of participants and prescribed medications.

Between 1 March 2012 and 31 August 2021, 1736 participants were enrolled into the EQUAL study. After excluding Swedish participants (*n* = 305), participants with missing or duplicated medication data (*n* = 54), and participants whose data were all recorded after their date of death (*n* = 6), 1371 participants remained. Of these, 808 did not die in the study follow up period and so were excluded, leaving 563 participants in the decedent cohort (see Fig. 1; for further detail see Supplementary data, Fig. S1). The baseline demographics of this population are shown in Table 1 (for comparison, baseline demographics and prescribing patterns are provided for the 808 non-decedent EQUAL participants in Supplementary data, Table S3). There were 174 (30.9%) female decedents, and 532 (94.5%) were white. At initial visit, median age was 77.8 years [interquartile range (IQR) 72.8–83.0] and median eGFR 18.1 mL/min/1.73 m^2^ (IQR 15.0–21.2).

**Table 1:** Baseline^a^ demographic and clinical characteristics of the analysed decedent cohort.

Characteristics		Decedents
Total	*n* (%)	563 (100.0)
Sex	Female, *n* (%)	174 (30.9)
Age	Years, median (IQR)	77.8 (72.8, 83.0)
eGFR^b^	mL/min/1.73 m^2^, median (IQR)	18.1 (15.0, 21.2)
Ethnicity	White, *n* (%)	532 (94.5)
BMI^c^	kg/m^2^, median (IQR)	28.1 (24.2, 31.4)
Country	Germany, *n* (%)	50 (8.9)
	Italy, *n* (%)	125 (22.2)
	The Netherlands, *n* (%)	98 (17.4)
	Poland, *n* (%)	32 (5.7)
	UK, *n* (%)	258 (45.8)
Primary renal disease	Diabetes mellitus, *n* (%)	119 (21.1)
	Glomerular disease, *n* (%)	36 (6.4)
	Hypertension/renal vascular disease, *n* (%)	200 (35.5)
	Miscellaneous renal disorders, *n* (%)	165 (29.3)
	Tubulointerstitial disease, *n* (%)	39 (6.9)
	Missing, *n* (%)	4 (0.7)
Educational attainment	No education, *n* (%)	9 (1.6)
	Primary school equivalent, *n* (%)	149 (26.5)
	Secondary school equivalent, *n* (%)	191 (33.9)
	University level equivalent, *n* (%)	55 (9.8)
	Missing, *n* (%)	159 (28.2)
Charlson Comorbidity Index	Mean (SD)	7.5 (1.8)
Comorbidity	Diabetes mellitus, *n* (%)	246 (43.7)
	Cerebrovascular disease, *n* (%)	92 (16.3)
	Peripheral vascular disease, *n* (%)	121 (21.5)
	Coronary artery disease, *n* (%)	172 (30.6)
	Hypertension, *n* (%)	464 (82.4)
Smoking status	Current smoker, *n* (%)	48 (8.5)
	Ex-smoker, *n* (%)	235 (41.7)
	Never smoker, *n* (%)	140 (24.9)
	Missing, *n* (%)	140 (24.9)
POMs	Number, mean (SD)	8.1 (3.0)
	Polypharmacy^d^, *n* (%)	491 (87.2)
	Hyperpolypharmacy^d^, *n* (%)	174 (30.9)

a At inclusion to EQUAL study.

b eGFR as calculated by the Modification of Diet in Renal Disease equation.

c Body mass index.

d Polypharmacy ≥5 medications; hyperpolypharmacy ≥10 medications

The 563 decedents had a total of 2793 study visits, with a median of 5 (IQR 3–7) study visits and median follow-up time of 2.2 years (IQR 1.1–3.8) pre-death. After applying medication exclusions, 22 173 individual POMs were analysed.

### Total number of prescribed oral medications

**Figure 2:**
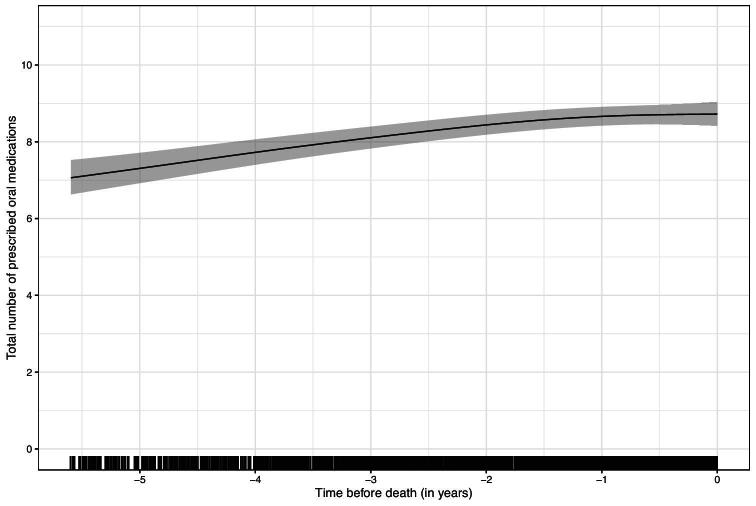
Total number of POMs over time leading up to death.

At their baseline visit, decedents were prescribed 8.1 [standard deviation (SD) 3.0] oral medications, with 87.2% experiencing polypharmacy (≥5 prescriptions). The total number of POMs rose throughout the analysed period (see Fig. 2) with 7.3 [95% confidence interval (CI) 6.9–7.7] prescriptions at 5 years pre-death, 8.3 (95% CI 8.0–8.6) at 2.5 years pre-death and 8.7 (95% CI 8.4–9.0) at death. Analysis of the difference in total number of POMs between each individuals’ first and last study visits—in those with >1 study visit (508/563, 90.2%)—showed no subgroups within the data (see Supplementary data, Fig. S2). At their final visit before death, 90.1% were experiencing polypharmacy.

In analyses stratified by sex, baseline Charlson Comorbidity Index and presence or absence of heart failure there was no difference in the total or trend in number of POMs over time (see Supplementary data, Fig. S3). Baseline age made no difference until the last 2 years pre-death, after which those who were older than the median age (77.8 years) were prescribed fewer oral medications than those who were younger. People with coronary artery disease had more POMs throughout the analysed period, as did people with diabetes, the latter's difference only partly attenuated by the exclusion of specific medications for the treatment of diabetes. Polish decedents had generally fewer POMs than decedents from other countries, but the overall trajectory of POMs was similar in all countries excepting Italy, which had no increase in number of POMs over the years approaching death. Participants who received dialysis during the studied period [179/563, 31.8% (610/2793, 21.8% study visits)] appeared to have similar numbers and trends of POMs to those who did not (see Supplementary data, Fig. S4).

### Target medication analyses

**Figure 3:**
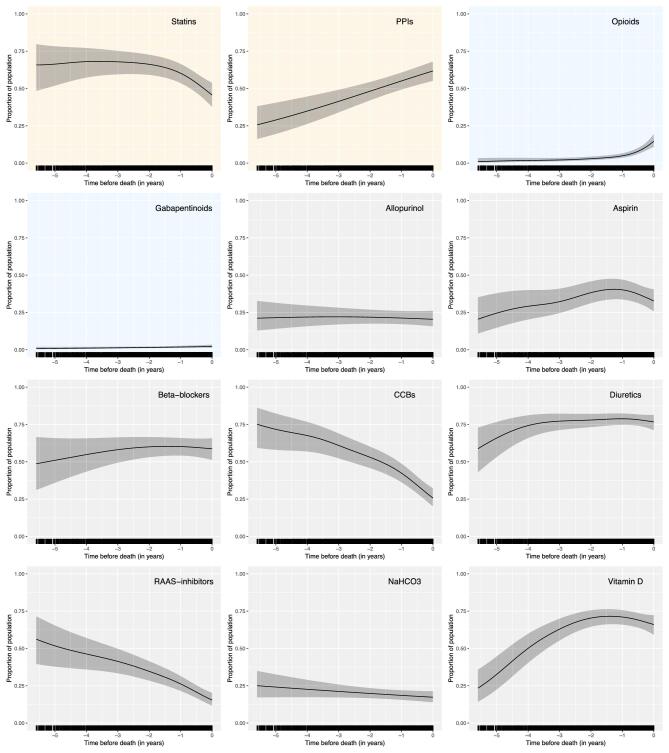
Medication trajectories over the time leading up to death. Each graph shows the proportion of the population prescribed the named medication class over time. Yellow—targets for deprescribing; blue—medications for symptom control; grey—others prescribed to >25% of the cohort. NaHCO3–sodium bicarbonate. Supplementary data, Table S2 shows the included compounds in each of these medication classes.

Specific medications identified as targets for deprescribing (see Fig. 3—yellow), those used for symptom control (see Fig. 3—blue) and those prescribed to >25% of the cohort (see Fig. 3—grey) were examined. In the last years of life, the proportion of people prescribed opioids and PPIs increased, the proportion prescribed statins, RAASis and CCBs decreased, and the proportion prescribed allopurinol, beta-blockers, diuretics, gabapentinoids and sodium bicarbonate remained stable. The proportion prescribed aspirin and vitamin D showed phasic patterns—increasing then levelling off.

**Figure 4:**
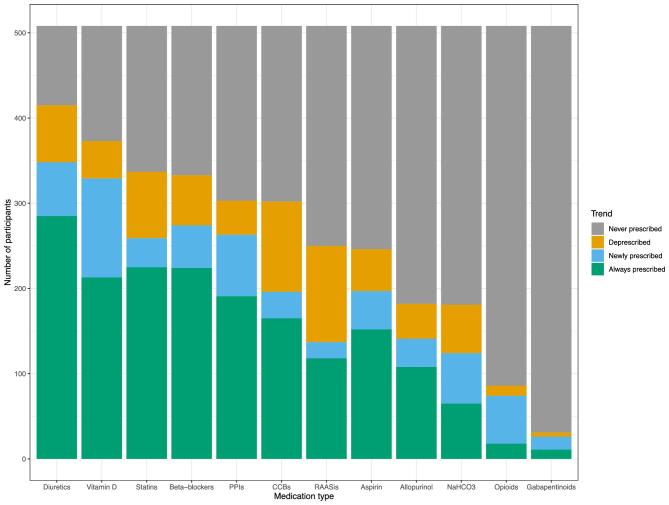
Trends of individuals’ prescribed medications comparing baseline study visits and final visits pre-death. NaHCO3–sodium bicarbonate. Supplementary data, Table S2 shows the included compounds in each of these medication classes.

Analysis of the differences between the POMs at individuals’ first and last study visits was performed on the 508/563 (90.2%) decedents who had >1 study visit (see Fig. 4). Over the whole analysed period, diuretics were the most common medication, prescribed to 81.7% (415/508) participants. Of those who were prescribed a statin at their initial visit, 25.7% (78/303) had had it stopped by their last visit; the same was true for 17.3% prescribed a PPI (40/231) (see Supplementary data, Fig. S5). At their first study visit, opioids were prescribed to 5.9% (30/508) participants and gabapentinoids to 3.1% (16/508); at their last study visit opioids were prescribed to 14.6% (74/508) participants and gabapentinoids to 5.1% (26/508).

### Sensitivity analyses

Limiting the analysed decedents to just those who had ≥3 study visits, and ≥1 year of data pre-death (391/563, 69.4% participants), did not make any difference to the total number or trajectory of POMs (see Supplementary data, Fig. S6). When including non-oral medications, participants were correspondingly prescribed a greater number of medications, and had a slightly steeper upwards trajectory over time when compared with the original analysis (see Supplementary data, Fig. S7).

## DISCUSSION

In this analysis of prescribing patterns for older people with advanced CKD, we have demonstrated that polypharmacy was highly prevalent and increased throughout the years leading up to death. Whilst the proportion of people prescribed certain medications decreased, this appeared to be outweighed at the population level by new prescriptions.

EQUAL decedents represent a group of people living with multiple long-term health conditions, for whom prescribing patterns reflect careful decisions to balance preventative and symptom-based care. People living with multiple long-term health conditions have a greater number of individual prescribers and a greater number of healthcare interactions, both known to increase the likelihood of polypharmacy, and reduce deprescribing [30]. This could partly explain why those with diabetes and CKD had more POMs than those without diabetes. We have also seen that older participants had a smaller increase in their number of POMs in the 2 years pre-death. They are more likely to have been living with frailty [31], a factor that increases the risk of negative outcomes when combined with polypharmacy [32], and one that may have reassured clinicians of the safety of deprescribing, given specific tools exist for this purpose [33].

One challenge facing prescribers is prognostication, which may help with the prioritization of symptom-directed vs preventative care. However, prognostication in CKD is not simple, and whilst tools to aid prognostication exist, performance metrics are low [34], and death in people with CKD can often be rapid and unexpected [35]. Nevertheless, some of the prescribing patterns revealed in this analysis are likely to reflect transitions towards less preventative, lower burden and more symptom-focussed care. We observed the proportion of people prescribed statins, CCBs and RAASis to decrease as death approached. Statins are a proposed target for deprescribing in the elderly with advanced CKD as specific data on their benefit are lacking in this group [15]. KDIGO recommends that a statin is prescribed to all people ≥50 years with an eGFR <60 mL/min/1.73 m^2^, provided they are not receiving dialysis [36]. However, large meta-analyses suggest that the benefits of statins become less clear in those with CKD stage G5 and those on dialysis [37, 38]. Discontinuation of statins in those predicted to be in their last year of life has been shown not to be associated with increased rates of mortality or major vascular events, but led to increased quality of life [39]. The decrease in CCBs and RAASis appeared more linear than the decrease in statins over the time analysed. This trend could be explained by responsive prescribing in the context of relaxed blood pressure (BP) targets or predicted prognosis, or reactive prescribing to naturally decreasing BP as people age, and approach the end of life. The latter fits with the terminal decline in BP that has been demonstrated in the EQUAL cohort previously [22], and other work hypothesizing that falling BP may be considered a physiological predictor of mortality in the elderly [40]. Although international guidelines suggest targeting a systolic BP <120 mmHg in people with CKD [41], it has been suggested that for older people a more relaxed target may be appropriate [16], to mitigate their increased risk of syncope and falls [42].

Diuretics and beta-blockers were more stable over time than the other antihypertensive classes, potentially due to preferential continuation of these medications for symptomatic benefit. Based on a previous study of EQUAL decedents [22], and others with advanced CKD [43], we know that as people approach death, their symptoms increase in number and severity. Within EQUAL, females experienced a higher symptom burden than males [44], but we observed sex making no difference to the numbers of POMs. The proportion prescribed PPIs increased towards end of life, a pattern which may reflect initiation for dyspepsia. However, PPIs may also be used preventatively, so this cannot be assumed. PPIs have been identified as a potential deprescribing target in older people with CKD [15] since they are often started for a short-term indication and not stopped, and long-term use is associated with increased risks of bone fracture, *Clostridium difficile* infection, pneumonia and acute kidney injury [45, 46]. The proportion of people prescribed opioids and gabapentinoids increased over the last years of life, almost certainly explained as treatment to target symptoms. Of the EQUAL cohort at baseline, >50% stated they were living with itch or pain [22, 44]; however, only 6.7% were prescribed gabapentinoids and 19.0% opioids at any time during the analysed period. These figures are likely to represent a combination of shared decision making between patients and clinicians, and undertreatment of symptoms. Recent international guidelines [21] have recommended that regular screening (preferably using a validated tool, e.g. IPOS: Renal [47]) be performed for all people with advanced CKD to detect and manage symptoms; and that this is part of their kidney supportive care needs regardless of their age, or their goals of treatment.

There is an increasing body of evidence looking at the potential benefits and practicalities of deprescribing, mostly in older, general populations [48]. Interventions in populations living with CKD have been successful at reducing the number of medications that a person is taking [17–19]; however, to truly gauge an intervention's effectiveness, multi-dimensional outcomes need to be measured. Recent expert consensus [49] suggests that alongside medication outcomes (e.g. number of pills) we also need to measure clinical outcomes (e.g. quality of life) and system outcomes (e.g. healthcare utilization).

Our work has provided valuable insight into how medicines are managed for this cohort of older people with advanced CKD in their final years of life. However, there are many questions that remain unanswered in the wider context of improving medicines management for people living with multimorbidity. How do we accurately distinguish appropriate from problematic polypharmacy [50]? How do we facilitate true shared decision-making in the clinic, when treatments are more complex, care is more fragmented and health-systems are increasingly time and resource constrained? Do nephrologists need to take more responsibility for medicines management, or should this be the domain of pharmacists, primary care physicians or geriatricians? Reeve *et* *al*. [14] have performed a large-scale thematic review of evidence around deprescribing, attempting to address these questions. Their paper provides insight into four components that might enable tailored deprescribing: an enabling infrastructure with clear multidisciplinary roles, consistent access to high-quality data to make prescribing decisions, tailored explanation to support a shared understanding between clinicians and patients, and continuity of care to enable ongoing monitoring and develop trust. Our data suggest that these considerations are likely to be relevant to nephrology practice, where polypharmacy is rife, and deprescribing not widespread. We believe that work at the level of the patient and prescriber is needed to understand how choices to start, continue and stop medications are being made for people with kidney disease. Knowing this will help to inform interventions that ensure prescribing is person-centered and aligned with each patient's disease and life course.

The main limitation of these analyses is the use of population-level data, meaning we cannot know the individual level reasons for the trends we have observed. Attempts to explore individual-level prescribing were limited by participants having varying amounts of time between their baseline and final visits pre-death, and by medication changes in between these visits not being captured. Data were not available to investigate whether prescribing patterns varied based on the location and size of the treating kidney unit, or whether the participant had access to a kidney supportive care service. It is worth noting that in the population-level analyses, the longer the length of time pre-death, the smaller the number of participants providing data. GAMs are flexible to this non-uniform data, and reassuringly in a sensitivity analysis where we excluded participants with <1 year and <3 study visits of data there were no differences in the observed trends. Medication data were only captured every 3–6 months, so it is possible that we are missing changes that occurred between study visits; especially relevant in the last year of life when medications often change more frequently. We also had no data on medication dose, pill number or medication adherence, which would have been useful to allow for further understanding—in particular we lacked knowledge of whether medications were being dose-reduced rather than withdrawn. Conducting medication analyses at the ‘class’ level (e.g. opioids) was felt to best capture whether deprescribing/prescribing was occurring in the last years of life, but we have not captured whether medications were switched within class (e.g. morphine to oxycodone), perhaps due to declining kidney function or the experience of adverse effects. Some participants are likely to have died shortly after follow-up was completed, and by limiting our analysis to known decedents these were excluded. Although this does not affect our conclusions, we have not been able to use the data that these participants may have added. Finally, all decedents had advanced CKD (recruited with incident eGFR <20 mL/min/1.73 m^2^) and the majority had died before the advent of the many new therapies for the treatment of kidney disease. There were no reported prescriptions for sodium-glucose cotransporter-2 inhibitors, non-steroidal mineralocorticoid receptor antagonists or glucagon-like peptide-1 agonists in our cohort. These drugs are revolutionizing treatment and outcomes for people with CKD, but future analyses are needed to determine the impact that these have on polypharmacy.

In summary, this descriptive analysis of elderly people with advanced CKD demonstrates that in the last years of life, polypharmacy is prevalent and continues to rise. To further understand these trends, we need to focus our work at the level of the individual patients, to see what mechanisms underlie prescribing, and to guide the design of future prescribing and deprescribing interventions.

## Supplementary Material

sfae301_Supplemental_Files

## Data Availability

Data from the EQUAL study are not publicly available. They may be requested from the EQUAL publication committee (contact: n.c.chesnaye@amsterdamumc.nl), by providing a potential study protocol and statistical analysis plan.
