# Supplementary material for: Prescribing patterns in older people with advanced chronic kidney disease towards the end of life
Source: Clin Kidney J. 2024 Oct 4;17(11):sfae301. doi: 10.1093/ckj/sfae301 (PMC11635369; doi:10.1093/ckj/sfae301)
Supplement: sfae301_Supplemental_Files [file sfae301_Supplemental_Files.zip › Supplementary figure 1 - Detailed cohort flow diagram.pptx]

## Slide 1
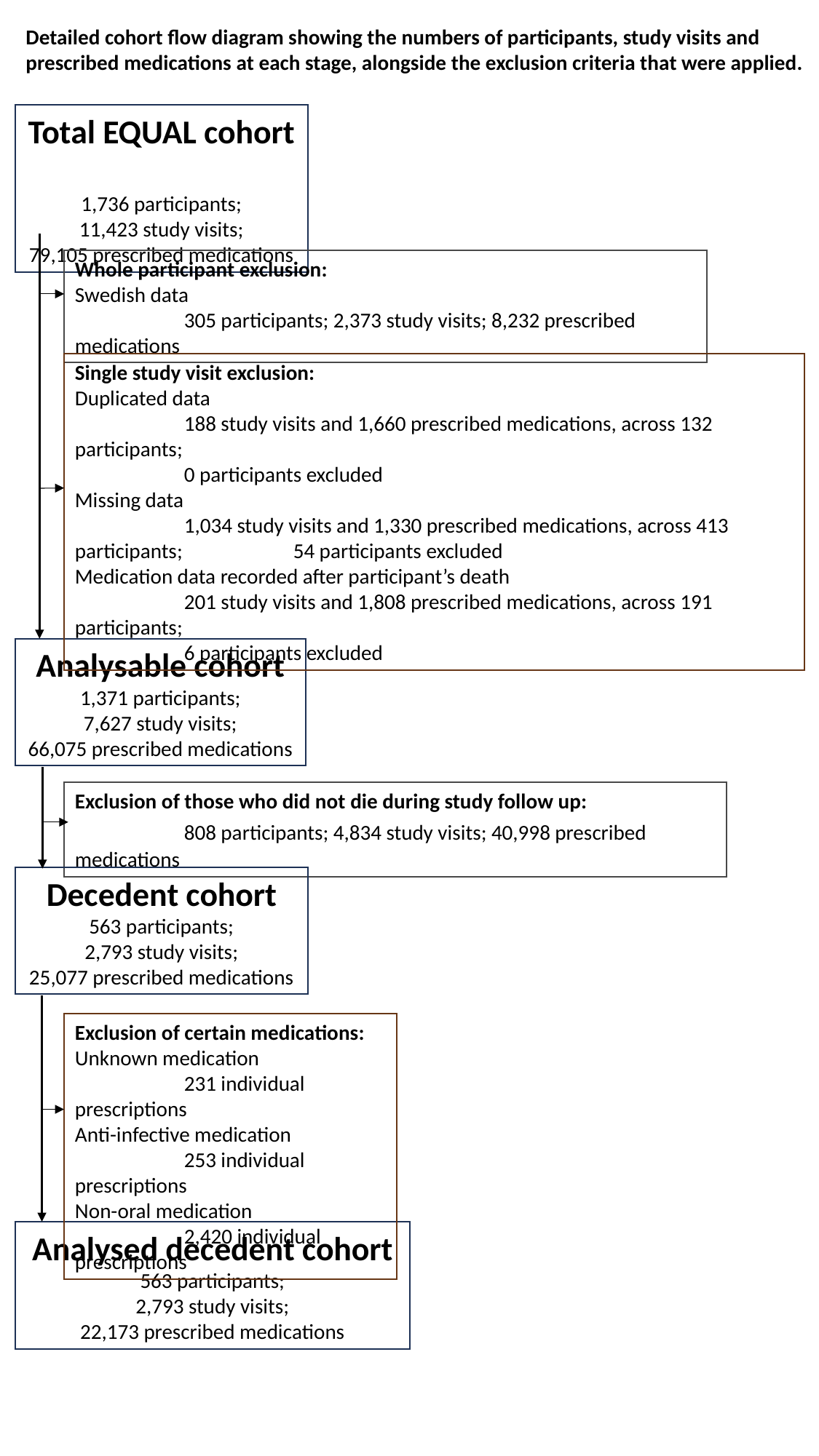

Detailed cohort flow diagram showing the numbers of participants, study visits and prescribed medications at each stage, alongside the exclusion criteria that were applied.
Total EQUAL cohort
1,736 participants;
11,423 study visits;
79,105 prescribed medications
Whole participant exclusion:
Swedish data
	305 participants; 2,373 study visits; 8,232 prescribed medications
Single study visit exclusion:
Duplicated data
	188 study visits and 1,660 prescribed medications, across 132 participants;
	0 participants excluded
Missing data
	1,034 study visits and 1,330 prescribed medications, across 413 participants; 	54 participants excluded
Medication data recorded after participant’s death
	201 study visits and 1,808 prescribed medications, across 191 participants;
	6 participants excluded
Analysable cohort
1,371 participants;
7,627 study visits;
66,075 prescribed medications
Exclusion of those who did not die during study follow up:
	808 participants; 4,834 study visits; 40,998 prescribed medications)
Decedent cohort
563 participants;
2,793 study visits;
25,077 prescribed medications
Exclusion of certain medications:
Unknown medication
	231 individual prescriptions
Anti-infective medication
	253 individual prescriptions
Non-oral medication
	2,420 individual prescriptions
Analysed decedent cohort
563 participants;
2,793 study visits;
22,173 prescribed medications
