# Supplementary material for: Prescribing patterns in older people with advanced chronic kidney disease towards the end of life
Source: Clin Kidney J. 2024 Oct 4;17(11):sfae301. doi: 10.1093/ckj/sfae301 (PMC11635369; doi:10.1093/ckj/sfae301)
Supplement: sfae301_Supplemental_Files [file sfae301_Supplemental_Files.zip › Supplementary figure 2 - Change in number of POMs, first to last visit.pdf]

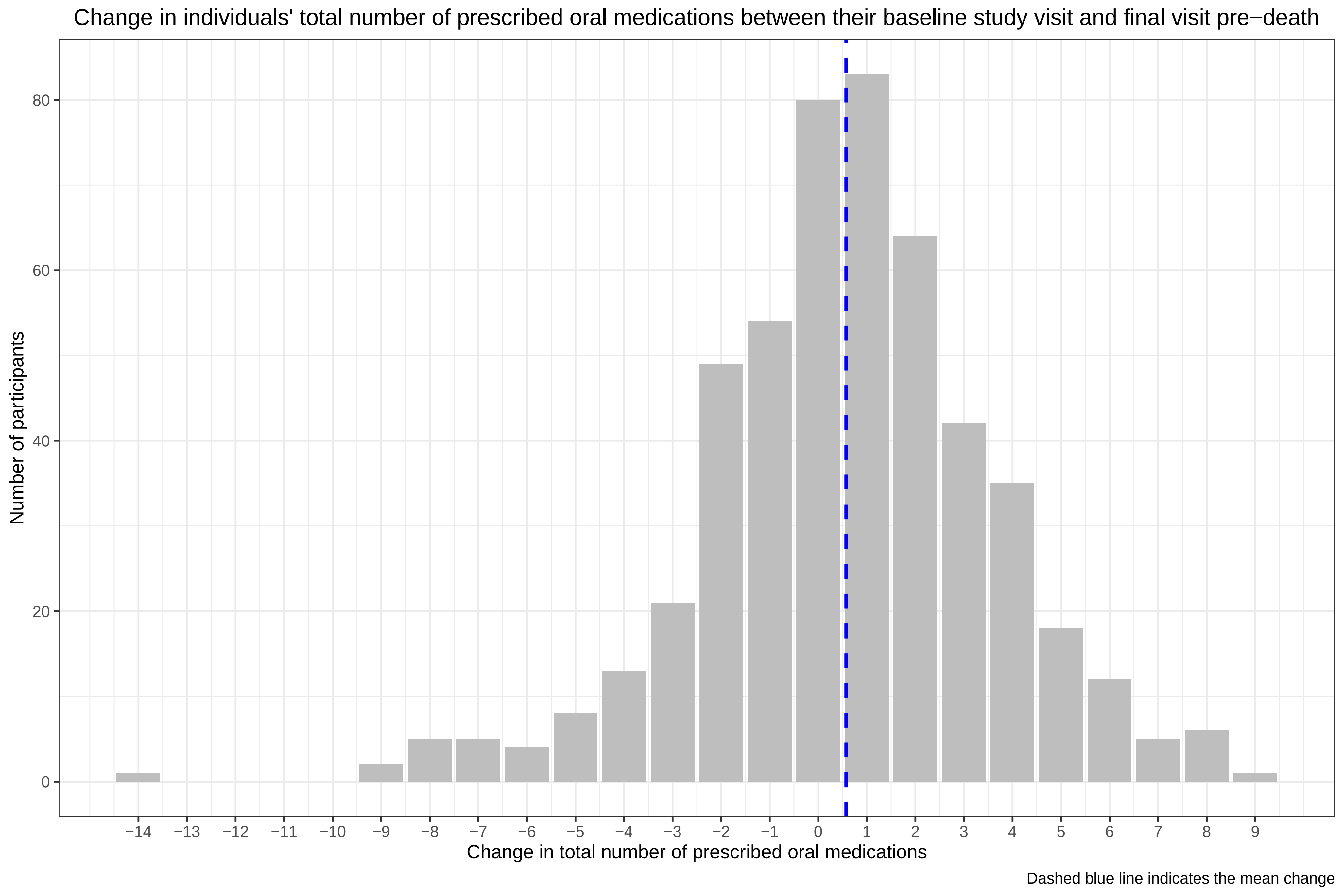

Change in individuals' total number of prescribed oral medications between their baseline study visit and final visit pre−death
Number of participants
0
20
40
60
80
−14
−13
−12
−11
−10
−9
−8
−7
−6
−5
−4
−3
−2
−1
0
1
2
3
4
5
6
7
8
9
Change in total number of prescribed oral medications
Dashed blue line indicates the mean change
