# Supplementary material for: Prescribing patterns in older people with advanced chronic kidney disease towards the end of life
Source: Clin Kidney J. 2024 Oct 4;17(11):sfae301. doi: 10.1093/ckj/sfae301 (PMC11635369; doi:10.1093/ckj/sfae301)
Supplement: sfae301_Supplemental_Files [file sfae301_Supplemental_Files.zip › Supplementary figure 3 - Trajectory in number of POMs stratified by covariates.pdf]

Trajectories of the total number of POMs in the time leading up to death stratified by baseline covariates

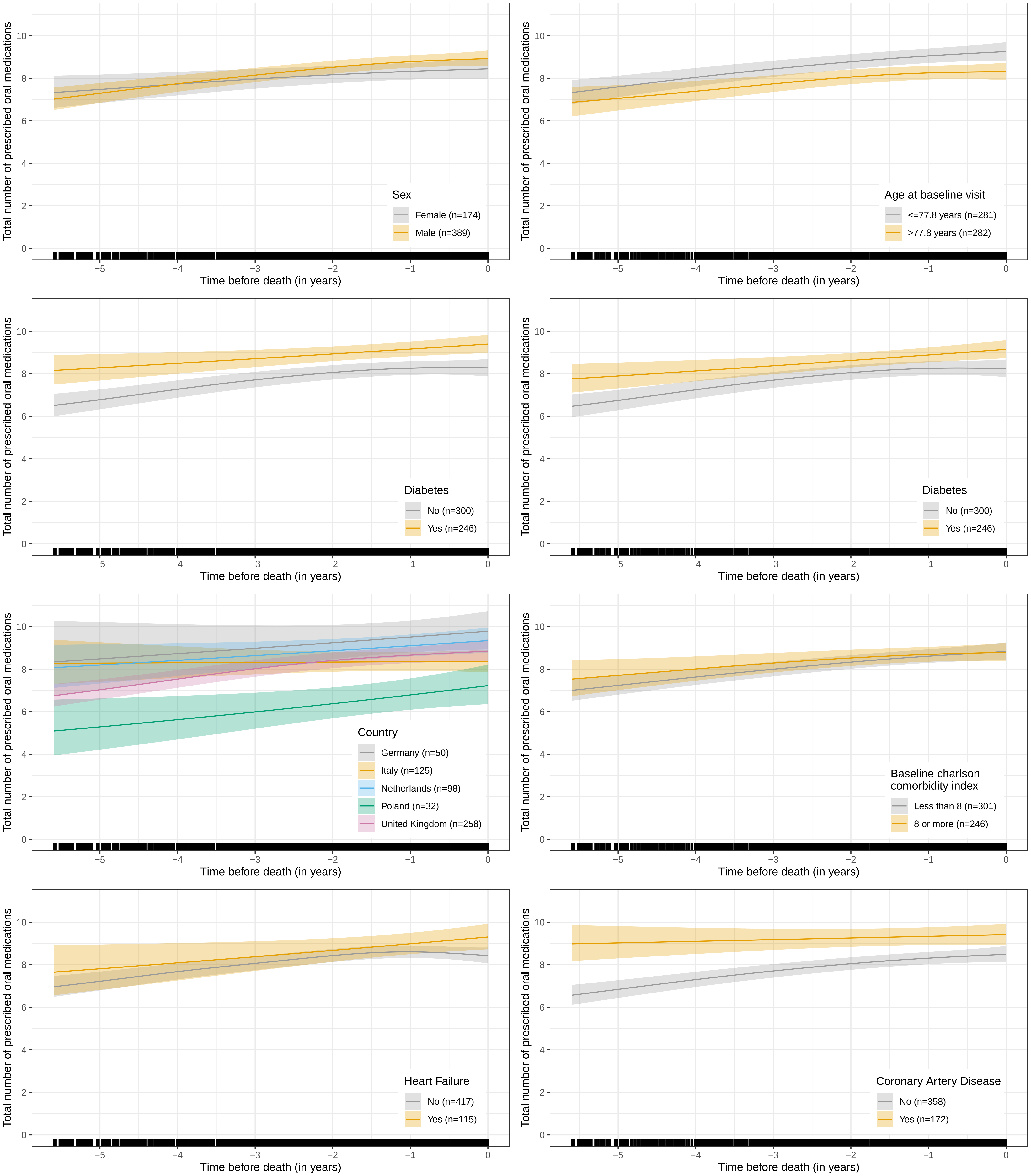

Numbers in each subgroup shown in each legend

top row left – sex (missing, n=0); top row right – age at baseline visit (missing, n=0);

second row left – presence of diabetes (missing, n=17); second row right – presence of diabetes (missing, n=17, all medications for the specific treatment of diabetes excluded);

third row left – country of origin (missing, n=0); third row right – baseline Charlson comorbidity index (missing, n=16);

bottom row left – presence of heart failure (missing, n=31); bottom row right – presence of coronary artery disease (missing, n=33).
