# Supplementary material for: Prescribing patterns in older people with advanced chronic kidney disease towards the end of life
Source: Clin Kidney J. 2024 Oct 4;17(11):sfae301. doi: 10.1093/ckj/sfae301 (PMC11635369; doi:10.1093/ckj/sfae301)
Supplement: sfae301_Supplemental_Files [file sfae301_Supplemental_Files.zip › Supplementary figure 4 - Trajectory number of POMs stratified by dialysis.pdf]

Trajectories of the total number of POMs in the time leading up to death stratified by dialysis status

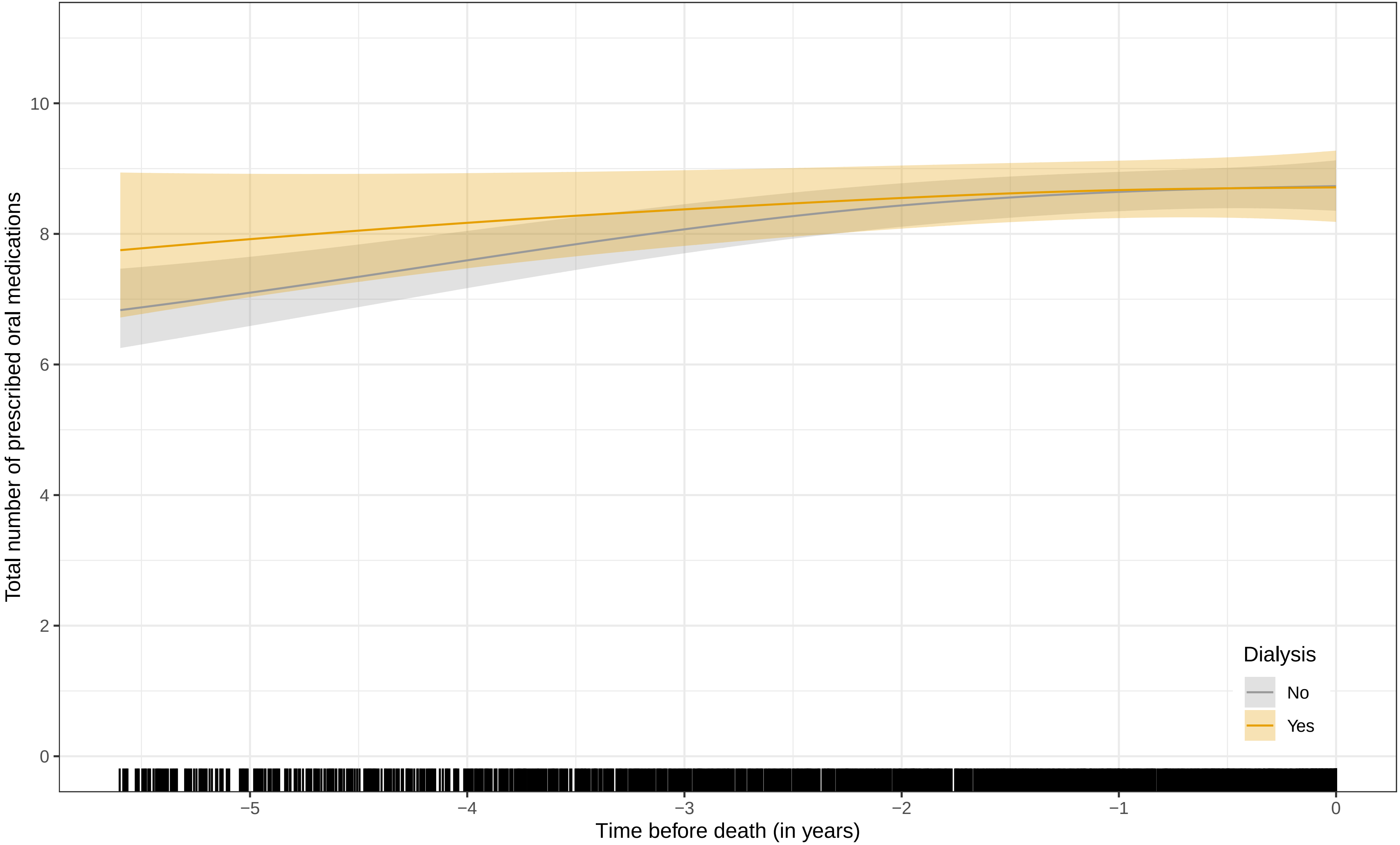

On entry to the EQUAL study, individuals were not in receipt of dialysis. 179/563 (31.8%) went on to start dialysis (haemodialysis or peritoneal dialysis) pre death.
In those that started dialysis, visits pre−dialysis were excluded from the above analysis, so that each individual only contributed to one or the other groups.
