# Supplementary material for: Prescribing patterns in older people with advanced chronic kidney disease towards the end of life
Source: Clin Kidney J. 2024 Oct 4;17(11):sfae301. doi: 10.1093/ckj/sfae301 (PMC11635369; doi:10.1093/ckj/sfae301)
Supplement: sfae301_Supplemental_Files [file sfae301_Supplemental_Files.zip › Supplementary figure 5 - Proportion decedents who had meds deprescribed.pdf]

Graph showing the proportions of individuals who had had medications deprescribed by their final study visit pre−death

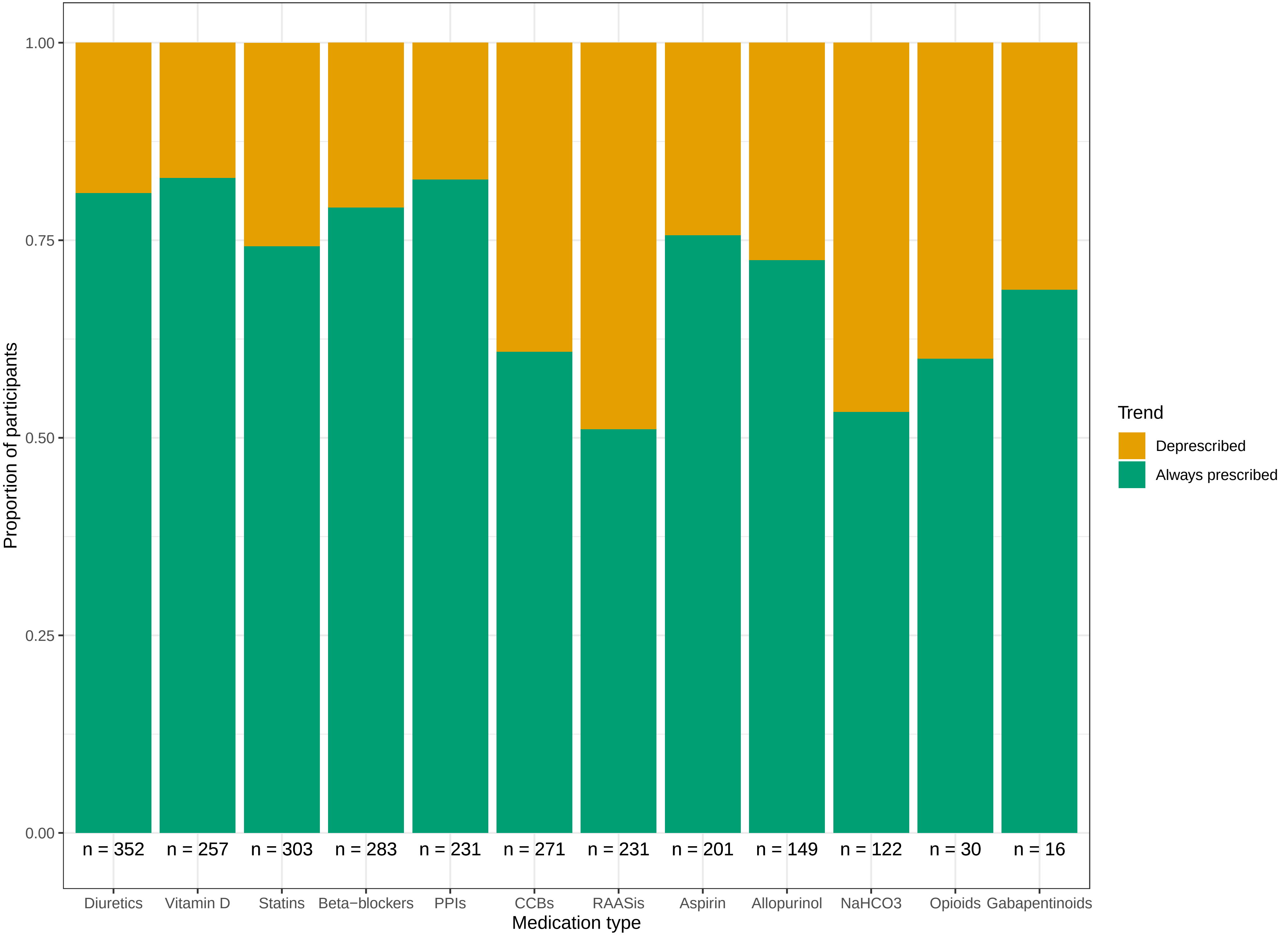

Only showing individuals who were prescribed each medication class at their initial study visit (numbers below each column).
'Always prescribed' − prescribed at initial study visit and final study visit pre−death; 'Deprescribed' − prescribed at initial study visit but not at final study visit pre−death.
Supplementary table 2 shows the included compounds in each of these medication classes.
