# Supplementary material for: Prescribing patterns in older people with advanced chronic kidney disease towards the end of life
Source: Clin Kidney J. 2024 Oct 4;17(11):sfae301. doi: 10.1093/ckj/sfae301 (PMC11635369; doi:10.1093/ckj/sfae301)
Supplement: sfae301_Supplemental_Files [file sfae301_Supplemental_Files.zip › Supplementary figure 6 - Sensitivity analysis 1 - 3+ visits, 1+ years data.pdf]

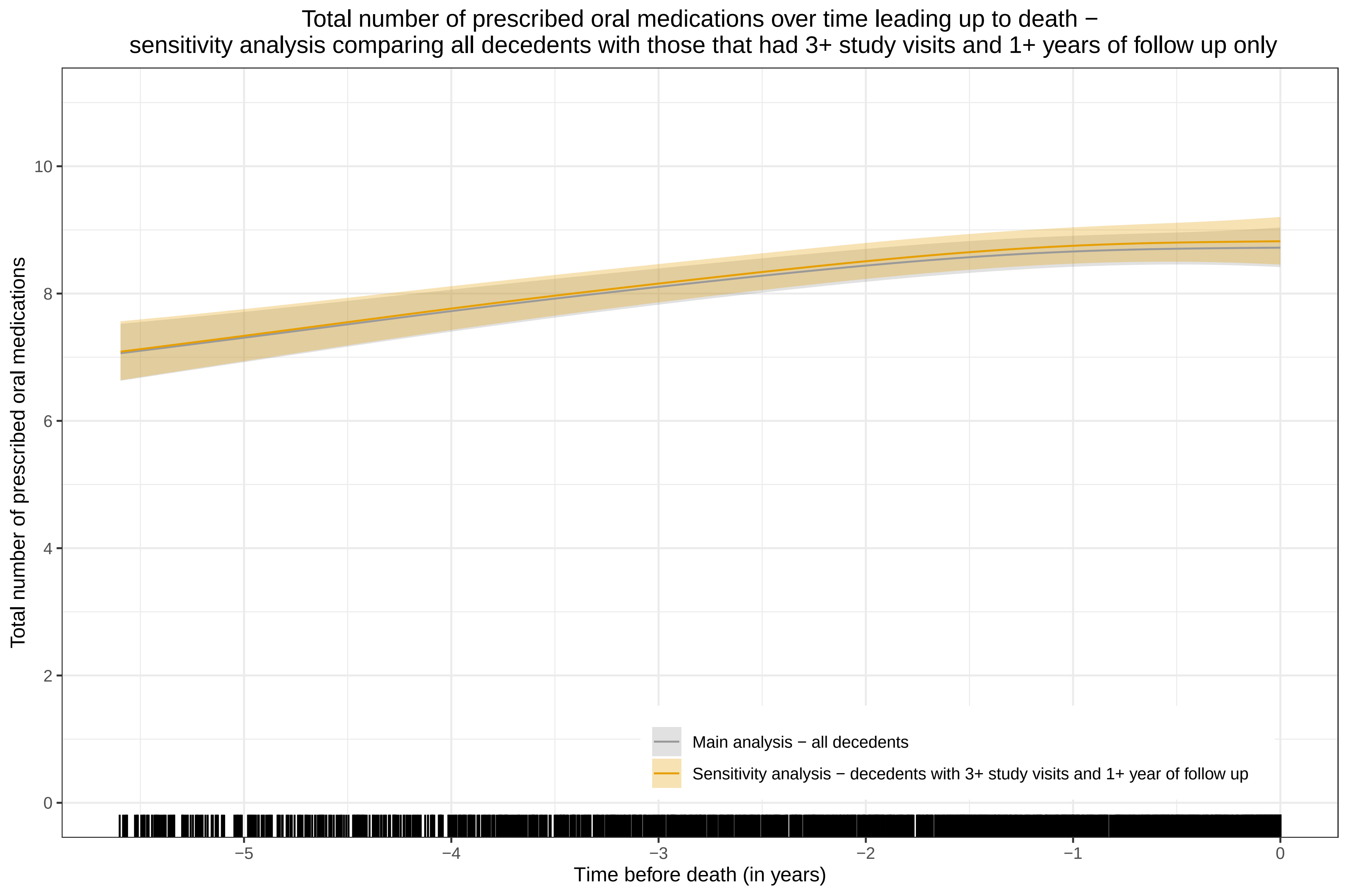
Total number of prescribed oral medications over time leading up to death −
sensitivity analysis comparing all decedents with those that had 3+ study visits and 1+ years of follow up only
Total number of prescribed oral medications
Time before death (in years)
10
8
6
4
2
0
−5
−4
−3
−2
−1
0
Main analysis − all decedents
Sensitivity analysis − decedents with 3+ study visits and 1+ year of follow up
