# Supplementary material for: Prescribing patterns in older people with advanced chronic kidney disease towards the end of life
Source: Clin Kidney J. 2024 Oct 4;17(11):sfae301. doi: 10.1093/ckj/sfae301 (PMC11635369; doi:10.1093/ckj/sfae301)
Supplement: sfae301_Supplemental_Files [file sfae301_Supplemental_Files.zip › Supplementary figure 7 - Sensitivity analysis 2 - Inc. non-oral medicines.pdf]

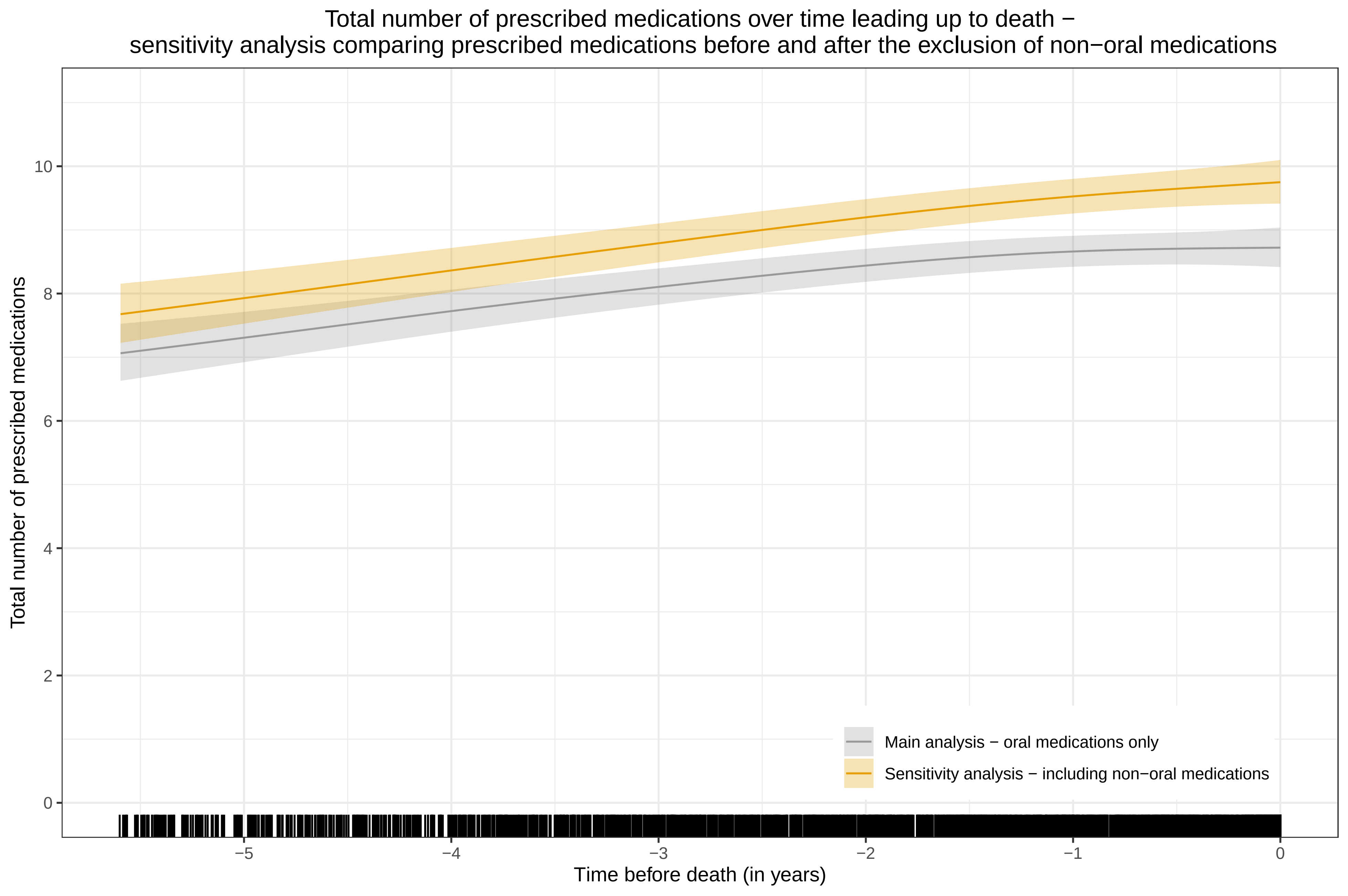
Total number of prescribed medications over time leading up to death −
sensitivity analysis comparing prescribed medications before and after the exclusion of non−oral medications
Total number of prescribed medications
10
8
6
4
2
0
−5
−4
−3
−2
−1
0
Time before death (in years)
Main analysis − oral medications only
Sensitivity analysis − including non−oral medications
